# A real-world pharmacovigilance study of neuroleptic malignant syndrome based on FDA adverse event reporting system

**DOI:** 10.3389/fphar.2024.1438661

**Published:** 2024-12-11

**Authors:** Yu Zhang, Wei Deng, Minjian Wang, Siying Luo, Song Li

**Affiliations:** ^1^ Department of Hospital Infection Control, Chongqing Mental Health Center, Chongqing, China; ^2^ Nursing Department, Chongqing Mental Health Center, Chongqing, China; ^3^ Department of Children and Adolescents, Chongqing Mental Health Center, Chongqing, China; ^4^ Sleep Medicine Center, Chongqing Mental Health Center, Chongqing, China

**Keywords:** neuroleptic malignant syndrome, FAERS, disproportionality analysis, pharmacovigilance, adverse event reports

## Abstract

**Background:**

Neuroleptic malignant syndrome (NMS) is a rare but potentially life-threatening adverse drug reaction. This study aims to identify the most prevalent drugs associated with the risk of NMS according to the United States Food and Drug Administration (FDA) Adverse Event Reporting System (FAERS) database.

**Methods:**

Analyses were performed using data from the FAERS database from January 2004 to June 2024. Single-drug signals were evaluated using the reporting odds ratio (ROR), proportional reporting ratio (PRR), information component (IC), and empirical Bayes geometric mean (EBGM). Meanwhile, comparisons were performed with drug labels. Additionally, subgroup analysis was conducted, focusing on adverse drug reaction signals among populations of different genders and age groups.

**Results:**

A total of 10,433 adverse event reports related to NMS were identified, with the top 50 drugs ranked by ROR mainly involving antipsychotics (18, 36%), antiparkinson drugs (10, 20%), antidepressants (7, 14%), antiepileptics (3, 6%), anxiolytics (3, 6%), as well as hypnotics and sedatives (3, 6%). NMS is more prevalent in males (5,713, 54.76%). Among the top 20 drugs with the strongest signal strength, the pediatric group showed an additional presence of benzodiazepines and antiepileptic drugs compared to the adult group.

**Conclusion:**

The current comprehensive pharmacovigilance study identified more drugs associated with NMS and provides references to clinicians for clinical practice. Also, further research is needed to investigate the causal relationship between these drugs and NMS.

## 1 Introduction

Neuroleptic malignant syndrome (NMS) is a potentially life-threatening adverse drug reaction to dopamine antagonists, characterized by hyperthermia, rigidity, altered mental status, autonomic dysfunction (i.e., diaphoresis, tachycardia, tachypnea, and labile blood pressure), as well as elevated creatine kinase and white blood cell count. In antipsychotic users, the incidence of NMS ranges from 0.06% to 1.4%, with mortality as high as 7.6% (Lao et al., 2020; Pileggi and Cook, 2016). The pathophysiology is not fully known, but there is a consensus that the use of dopamine receptor antagonists leads to the blockade of dopamine D2 signaling or related pathways in the substantia nigra-striatum, hypothalamus, and cortex, resulting in neurological dysfunction. It can be caused by all classes of antipsychotic drugs and other drugs that might also block dopamine receptors, such as antihistaminergic antiemetics. Beyond that, there are also reports of lithium salts, carbamazepine, and antidepressants causing NMS (Patil et al., 2016). The complications of NMS are common causes of death in critically ill patients, including rhabdomyolysis, renal failure, cardiac arrhythmias, circulatory collapse, and disseminated intravascular coagulation (DIC). With the increasing incidence of mental disorders and the widespread use of antipsychotics and non-antipsychotics, reports of NMS are gradually increasing. NMS is often misdiagnosed, lacks specific treatments, and has a high mortality rate, with the key to treatment lying in early drug cessation. Therefore, being familiar with the adverse reactions of high-risk medications in clinical practice is crucial for preventing the occurrence of NMS.

The majority of case reports concerning NMS typically involve both typical and atypical antipsychotics, occasionally including antidepressants, antiepileptic drugs, etc., It’s important to note that information regarding the potential risk of NMS with specific medications largely stems from case reports, as conducting clinical randomized controlled trials is challenging due to the rarity of NMS. Additionally, there are limited retrospective observational studies on this topic. As one of the largest pharmacovigilance databases, the U.S. Food and Drug Administration (FDA) Adverse Event Reporting System (FAERS) database has played a major role in the evaluation of drug safety. Based on safety signals obtained by data mining using the post-marketing surveillance database, it is possible to detect unknown adverse events (AEs) that have not been discovered in clinical trials as well as evaluate safety in specific populations and reflect actual clinical uses. Currently, only a few pharmacovigilance studies on NMS are conducted based on Japanese populations, and data from other regions is lacking. Therefore, this study aims to analyze drugs associated with NMS occurrence based on the FAERS database and provide evidence for the selection of clinical drugs.

## 2 Materials and methods

### 2.1 Data collection

This retrospective pharmacovigilance study extracted data from the FAERS database, which contains demographic information, drug information, and reaction information. For this study, the AEs of NMS were searched from the first quarter of 2004 to the second quarter of 2024. The patient’s information, including demographic and administrative data, drug and therapy data, and reporting sources, was collected. We searched the FAERS database by adopting the preferred term (PT) “Neuroleptic malignant syndrome (PT code: 10029282)” according to the Medical Dictionary for Regulatory Activities (MedDRA) version 26.1. There are four classifications to group each case according to the role of the medications administered in the adverse events: primary suspect drug (PS), secondary suspect drug (SS), concomitant (C), and interaction (I). We extracted data for every case that received the designation of PS.

In this study, duplicate reports that described the same adverse medication occurrence in the same patient were eliminated. Because the data used in the current study were de-identified and publicly available from the FAERS website, the study was exempt from ethical review.

### 2.2 Statistical analysis

The R software, specifically version 4.3.3, was used for data processing, statistical calculations, and visualization. A descriptive analysis was conducted to describe the clinical characteristics of NMS cases, including the patient’s gender, age, reporting country, and indications. The top 50 drugs related to NMS were selected based on the number of reports. The 50 drugs were classified according to the Anatomical Therapeutic Chemical (ATC) classification system.

Based on the contrast between observed and expected numbers of reports, disproportionality analysis was used to generate hypotheses on possible associations between drugs and AEs. To improve the results’ reliability, disproportionality analysis was carried out using the reported odds ratio (ROR), proportional reporting rate (PRR), information component (IC), and empirical Bayes geometric mean (EBGM) to detect the NMS risk signal for each drug and conducted calculations using a 2-by-2 contingency table (Supplementary Table S1). The Equation and Criteria of the above four methods are detailed in Supplementary Table S2. A larger value indicates a stronger signal value and a safety signal was considered when it met four algorithm criteria simultaneously. Following that, separate disproportionality analyses were performed based on gender and age.

## 3 Results

### 3.1 Characteristics of adverse event reports

**FIGURE 1:**
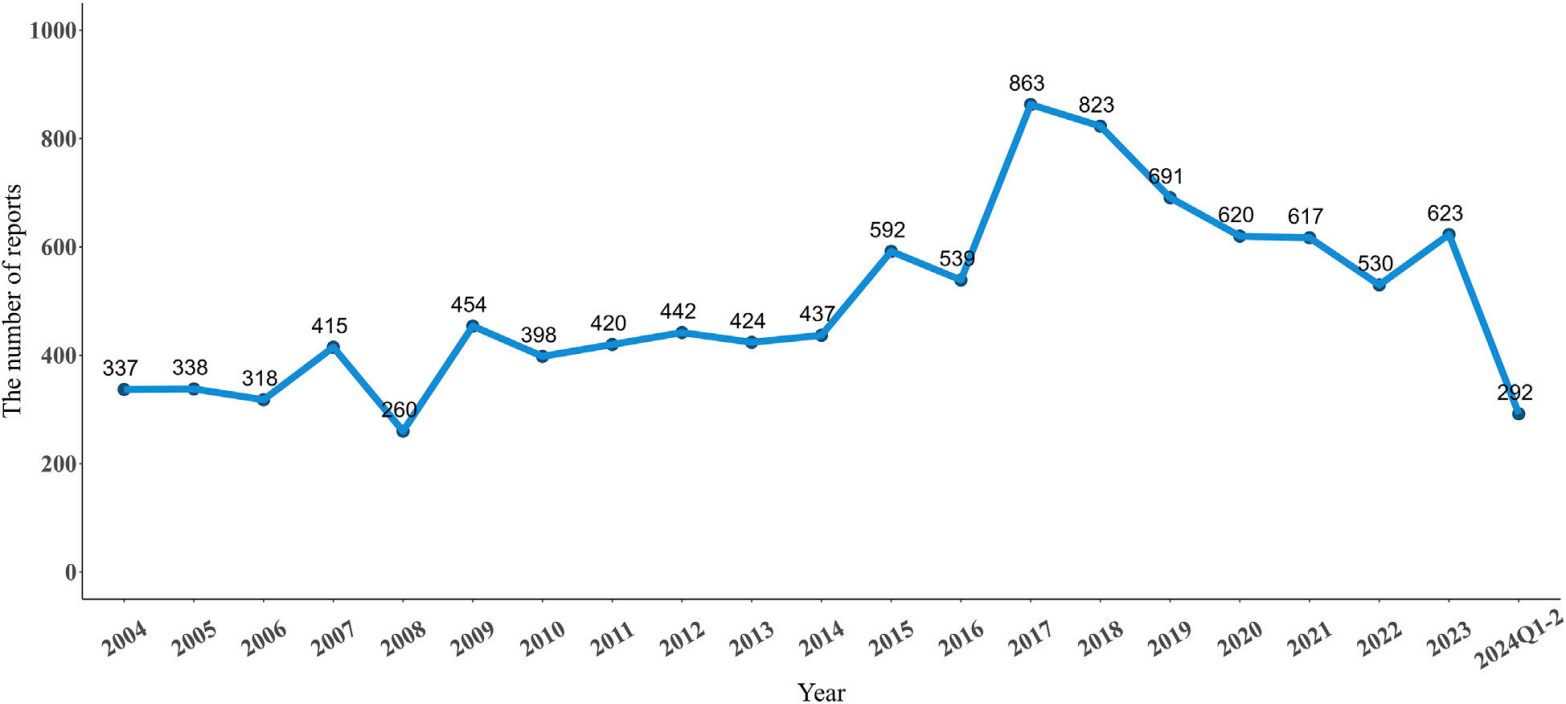
Annual neuroleptic malignant syndrome reports from Q1 2004 to Q2 2024.

From Q1 2004 to Q2 2024, there were 10,433 adverse event reports (AERs) in FAERS database reported for NMS. As shown in Figure 1, the number of reported NMS peaked in 2017 at 863 reports. Starting in 2018, the number began to decline, but the general trend from 2004 to 2024 shows an increase in volatility. The clinical characteristics of these 10,433 reports are listed in Table 1. The median age of the study population was 48 years (interquartile range 31–62). Excluding those of unknown age, the remaining cases were mainly in the 18–64 age group (57.42%). The number of reports from males (5,713, 54.76%) is higher than from females (3,780, 36.23%). The top five indications for drug use were: schizophrenia (1,985, 19.03%), bipolar disorder (836, 8.01%), depression (714, 6.84%), psychotic disorder (514, 5.56%), and Parkinson’s disease (280, 4.93%). The country with the highest number of reports was the United States (1,727, 16.55%), followed by the United Kingdom (942, 9.03%), Japan (923, 8.85%), France (446, 4.27%), and Canada (319, 3.06%).

**TABLE 1:** Clinical characteristics of reported neuroleptic malignant syndrome.

Characteristics	Reports, n (%)
Overall	10,433
Age, years	
Median, IQR	48.00 (31.00,62.00)
≤17	593 (5.68)
18–64	5,991 (57.42)
≥65	1,834 (17.58)
Unknow	2,015 (19.31)
Gender	
Female	3,780 (36.23)
Male	5,713 (54.76)
Unknown	940 (9.01)
Indications	
Schizophrenia	1,985 (19.03)
Unknown	1,875 (17.97)
Bipolar disorder	836 (8.01)
Depression	714 (6.84)
Psychotic disorder	514 (5.56)
Parkinson’s disease	280 (4.93)
Agitation	274 (2.63)
Dementia	105 (1.01)
Anxiety	90 (0.86)
Mania	87 (0.83)
Reported country	
United States	1,727 (16.55)
United Kingdom	942 (9.03)
Japan	923 (8.85)
France	446 (4.27)
Canada	319 (3.06)
Spain	269 (2.58)
India	209 (2.00)
Portugal	207 (1.98)
Germany	202 (1.94)
Italy	199 (1.91)
Time to onset, days	
Median, IQR	16.00 (3.00,137.00)

Abbreviations: IQR, interquartile range.

### 3.2 Disproportionality analysis

#### 3.2.1 Overall

**FIGURE 2:**
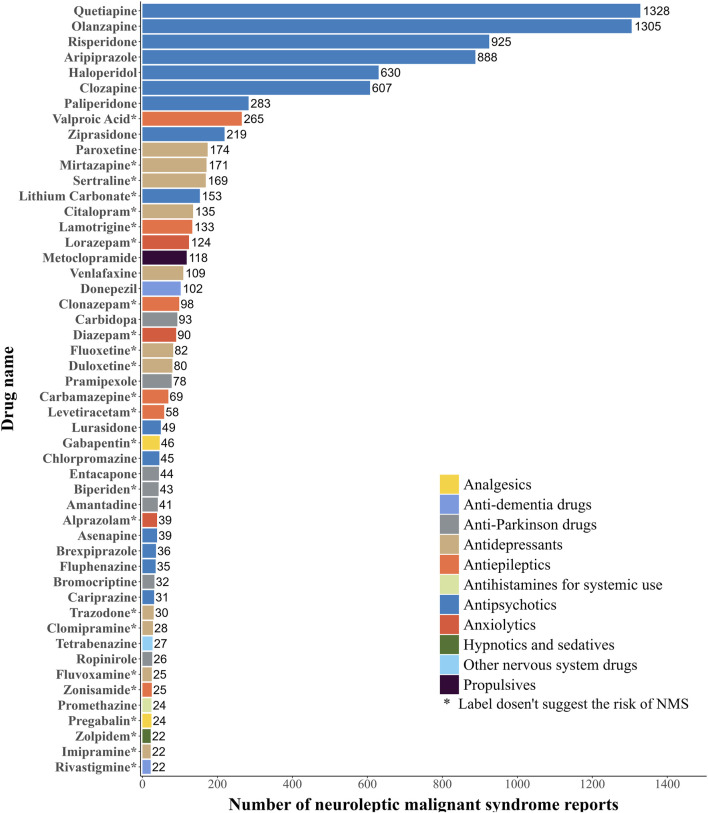
Top 50 drugs with the highest number of reported neuroleptic malignant syndrome.

The current study summarized the top 50 drugs ranked by the frequencies of AERs (Figure 2). These drugs were classified according to the World Health Organization (WHO) ATC system. As for the frequencies of AERs, quetiapine (1,328 reports) is the most frequently reported drug, followed by olanzapine (1,305 reports), risperidone (925 reports), aripiprazole (888 reports), haloperidol (630 reports), clozapine (607 reports), paliperidone (283 reports), valproic acid (265 reports), ziprasidone (219 reports) and paroxetine (174 reports). The main categories with a high number of drugs among these 50 were antipsychotics (15, 30%), antidepressants (11, 22%), antiparkinson drugs (7, 14%), antiepileptics (6, 12%), and anxiolytics (3, 6%). Of these 50 drugs, 24 drugs didn’t indicate NMS risk on the label, mainly including antidepressants (9, 37.5%) and antiepileptics (6, 25%), while the remaining 26 drugs did.

According to the signal strength, the top 50 drugs are listed in Table 2, all of which have statistically significant signal strengths. The results of ROR, PRR, IC, and EBGM are consistent. The top 5 drugs ranked by ROR were: favipiravir (ROR 1727.47, 95%CI 431.97-6908.26), biperiden (ROR 348.41, 95%CI 250.95-483.71), amisulpride (ROR 216.12, 95%CI 121.38-384.80), trihexyphenidyl (ROR 149.84, 95%CI 84.99-264.17), and fluphenazine (ROR 145.77, 95%CI 103.19-205.93). According to drug classification, the most common type of drugs is antipsychotics (18, 36%), followed by antiparkinson drugs (10, 20%), antidepressants (7, 14%), antiepileptics (3, 6%), anxiolytics (3, 6%), and hypnotics and sedatives (3, 6%). Of the top 50 drugs, 28 drugs indicate NMS risk on the label, mainly including antipsychotics (17, 60.7%) and antiparkinson drugs (7, 25%), while the other 22 drugs did not.

**TABLE 2:** Signal strength for drugs associated with neuroleptic malignant syndrome.

WHO ATC category	Drug name	Case reports	ROR (95% CI)	PRR (95% CI)	IC(IC025)	EBGM(EBGM05)
Antipsychotics	Amisulpride	13	216.12 (121.38, 384.8)	192.22 (115.47, 319.97)	7.58 (6.79)	191.98 (118.47)
	Fluphenazine	35	145.77 (103.19, 205.93)	134.53 (98.32, 184.08)	7.07 (6.58)	134.09 (100.42)
	Loxapine	6	124.9 (54.53, 286.09)	116.55 (54.27, 250.32)	6.86 (5.75)	116.48 (58.22)
	Chlorpromazine	45	122.13 (90.21, 165.34)	114.16 (86.76, 150.2)	6.83 (6.4)	113.67 (88.22)
	Haloperidol	630	102.26 (94.15, 111.07)	96.92 (89.61, 104.82)	6.51 (6.39)	91.13 (85.04)
	Olanzapine	1,305	55.64 (52.46, 59)	54.13 (51.04, 57.41)	5.57 (5.49)	47.49 (45.21)
	Ziprasidone	219	40.94 (35.75, 46.87)	40.03 (34.9, 45.92)	5.29 (5.1)	39.21 (35.01)
	Lithium Carbonate	153	39.66 (33.74, 46.6)	38.8 (33.17, 45.39)	5.26 (5.03)	38.25 (33.41)
	Perphenazine	5	38.39 (15.82, 93.15)	37.58 (15.86, 89.02)	5.23 (4.06)	37.56 (17.89)
	Quetiapine	1,328	34.82 (32.85, 36.9)	34.23 (32.28, 36.3)	4.91 (4.82)	30 (28.58)
	Prochlorperazine	10	32.8 (17.54, 61.33)	32.21 (17.54, 59.14)	5.01 (4.15)	32.18 (19.06)
	Aripiprazole	888	25.01 (23.34, 26.8)	24.69 (23.28, 26.19)	4.5 (4.4)	22.68 (21.4)
	Risperidone	925	24.17 (22.58, 25.86)	23.87 (22.51, 25.32)	4.45 (4.35)	21.84 (20.64)
	Paliperidone	283	14.46 (12.84, 16.28)	14.35 (12.76, 16.14)	3.81 (3.64)	13.99 (12.67)
	Lumateperone	22	13.05 (8.58, 19.86)	12.96 (8.59, 19.56)	3.69 (3.1)	12.94 (9.11)
	Clozapine	607	11.53 (10.62, 12.51)	11.46 (10.6, 12.39)	3.44 (3.32)	10.85 (10.13)
	Cariprazine	31	11.49 (8.06, 16.36)	11.42 (8.03, 16.25)	3.51 (3.01)	11.39 (8.47)
	Asenapine	39	10.01 (7.31, 13.73)	9.96 (7.28, 13.63)	3.31 (2.86)	9.93 (7.63)
Antidepressants	Maprotiline	11	120.35 (65.29, 221.82)	112.58 (63.77, 198.75)	6.81 (5.97)	112.46 (67.42)
	Imipramine	22	45.21 (29.6, 69.07)	44.09 (29.21, 66.54)	5.46 (4.86)	43.99 (30.86)
	Clomipramine	28	40.84 (28.06, 59.44)	39.92 (27.51, 57.93)	5.32 (4.78)	39.82 (29.09)
	Fluvoxamine	25	34.18 (23, 50.8)	33.54 (22.66, 49.64)	5.06 (4.5)	33.46 (24.02)
	Mirtazapine	171	16.67 (14.32, 19.4)	16.52 (14.12, 19.32)	4.02 (3.81)	16.27 (14.33)
	Trazodone	30	9.85 (6.87, 14.1)	9.8 (6.89, 13.95)	3.29 (2.78)	9.77 (7.23)
	Paroxetine	174	7.88 (6.78, 9.16)	7.85 (6.71, 9.18)	2.95 (2.74)	7.74 (6.82)
Antiparkinson drugs	Biperiden	43	348.41 (250.95, 483.71)	290.28 (220.62, 381.93)	8.18 (7.71)	289.09 (219.68)
	Trihexyphenidyl	13	149.84 (84.99, 264.17)	137.97 (81.27, 234.21)	7.11 (6.32)	137.8 (85.75)
	Bromocriptine	32	53.19 (37.4, 75.65)	51.64 (37.01, 72.06)	5.69 (5.19)	51.48 (38.34)
	Entacapone	44	45.63 (33.81, 61.59)	44.49 (33.16, 59.7)	5.47 (5.04)	44.3 (34.47)
	Amantadine	41	24.98 (18.34, 34.02)	24.64 (18.01, 33.72)	4.62 (4.18)	24.55 (18.96)
	Benztropine	6	23.35 (10.43, 52.26)	23.05 (10.32, 51.48)	4.53 (3.45)	23.04 (11.74)
	Pergolide	4	22.08 (8.23, 59.2)	21.81 (8.19, 58.11)	4.45 (3.17)	21.8 (9.55)
	Pramipexole	78	15.81 (12.64, 19.77)	15.67 (12.63, 19.44)	3.96 (3.64)	15.56 (12.91)
	Cabergoline	20	11.57 (7.45, 17.97)	11.5 (7.47, 17.7)	3.52 (2.9)	11.48 (7.94)
	Stalevo 100 (levodopa/carbidopa/entacapon)	6	8.73 (3.91, 19.48)	8.69 (3.89, 19.41)	3.12 (2.05)	8.69 (4.44)
Antiepileptics	Valproic Acid	265	14.06 (12.44, 15.9)	13.96 (12.41, 15.7)	3.77 (3.59)	13.63 (12.3)
	Zonisamide	25	13.22 (8.91, 19.6)	13.13 (8.87, 19.43)	3.71 (3.15)	13.1 (9.42)
	Clonazepam	98	8.89 (7.29, 10.86)	8.85 (7.27, 10.77)	3.13 (2.85)	8.78 (7.43)
Anxiolytics	Lorazepam	124	14.75 (12.34, 17.61)	14.63 (12.26, 17.45)	3.85 (3.6)	14.47 (12.47)
	Buspirone	13	12.39 (7.18, 21.39)	12.31 (7.11, 21.31)	3.62 (2.86)	12.29 (7.79)
	Diazepam	90	9.2 (7.47, 11.33)	9.16 (7.38, 11.36)	3.18 (2.89)	9.09 (7.64)
Hyponotics and Sedatives	Dexmedetomidine	15	12.11 (7.29, 20.14)	12.04 (7.23, 20.04)	3.59 (2.88)	12.02 (7.86)
	Lemborexant	4	10.95 (4.1, 29.27)	10.89 (4.09, 29.02)	3.44 (2.17)	10.88 (4.78)
	Triazolam	7	7.83 (3.72, 16.45)	7.8 (3.7, 16.43)	2.96 (1.96)	7.79 (4.19)
Antivirals for systemic use	Favipiravir	4	1727.47 (431.97, 6908.26)	864.23 (435.21, 1716.15)	9.75 (8.18)	863.9 (270.88)
Anti-dementia drugs	Donepezil	102	20.37 (16.74, 24.78)	20.15 (16.56, 24.51)	4.32 (4.04)	19.96 (16.94)
Antihistamines for systemic use	Promethazine	24	11.76 (7.87, 17.57)	11.69 (7.9, 17.3)	3.54 (2.98)	11.66 (8.33)
Muscle relaxants	Dantrolene	5	31.3 (12.92, 75.81)	30.76 (12.99, 72.87)	4.94 (3.78)	30.74 (14.67)
Drugs for functional gastrointestinal disorders	Dicyclomine	4	12.25 (4.58, 32.76)	12.17 (4.57, 32.43)	3.6 (2.33)	12.17 (5.34)
Propulsives	Metoclopramide	118	9.5 (7.92, 11.4)	9.45 (7.92, 11.27)	3.23 (2.96)	9.36 (8.04)

Abbreviations: ROR, reporting odds ratio; PRR, proportional reporting ratio; IC, information component; EBGM, empirical Bayes geometric mean; CI, confidence interval.

#### 3.2.2 Subgroup analysis

**FIGURE 3:**
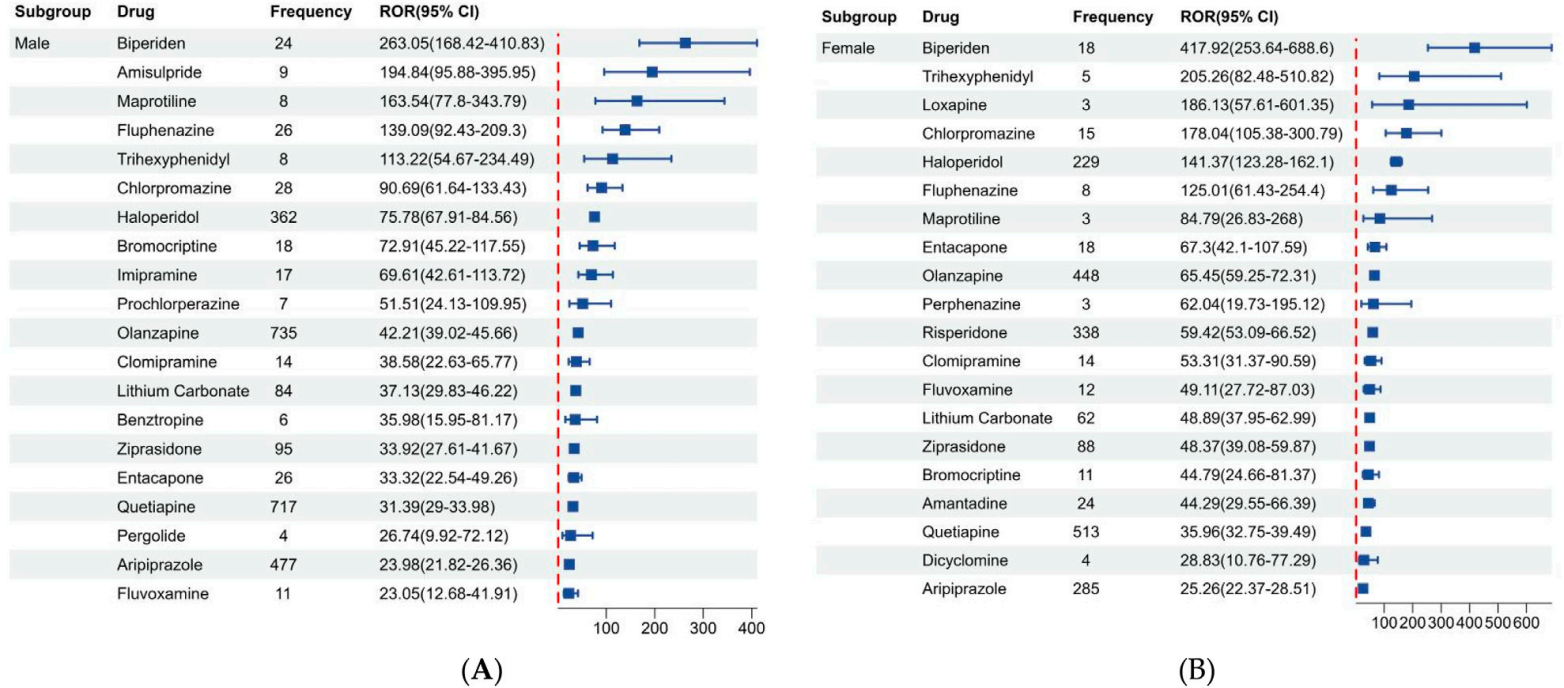
Signals detection among different gender groups. **(A)** Male group. **(B)** Female group.

Figure 3 shows the disproportionality results based on gender, we listed the top 20 drugs related to NMS in males and females based on ROR values. Of the 10,433 reports associated with NMS, 9,493 reported known gender and were divided into male (5,713, 54.76%) and female (3,780, 36.23%) groups. Additionally, among the top 20 drugs in different genders, antipsychotics, antidepressants, and antiparkinson drugs were predominant. Biperiden, amisulpride, maprotiline, fluphenazine, and trihexyphenidyl are the top 5 drugs with high ROR in males. While biperiden, trihexyphenidyl, loxapine, chlorpromazine, and haloperidol are the top 5 drugs with high ROR in females.

**FIGURE 4:**
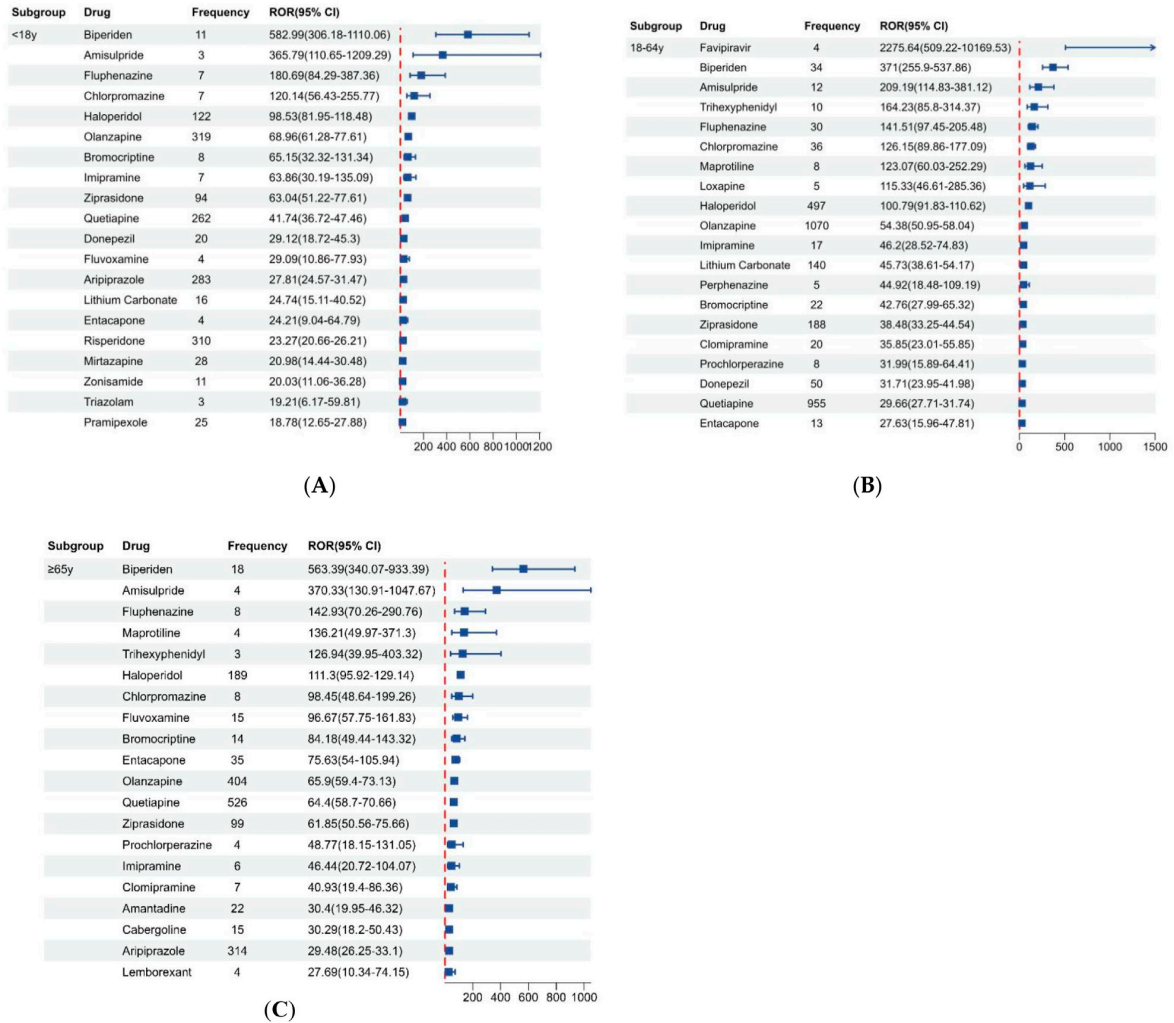
Signals detection based on patient age. **(A)** Age group under 18 years old. **(B)** Age group 18–64 years old. **(C)** Age group over 65 years old.

Figure 4 displays the disproportionality results based on age, we found that there are different types of risk drugs detected between the pediatric and adult groups. Besides antipsychotics, antiparkinson drugs, and antidepressants, the drugs with strong positive signals detected in patients under 18 years old included another antiepileptic (zonisamide) and benzodiazepine (triazolam). Notably, exclusive to the 18–65 years old group, favipiravir exhibited the highest signal strength (ROR 2275.64, 95% CI 509.22-10169.53).

## 4 Discussion

Based on all spontaneous adverse event reports in the FAERS database since 2004, the study comprehensively investigated drug safety signals associated with NMS occurrence. Through frequency and four disproportionality analysis methods, this study found that drugs primarily associated with NMS include antipsychotics, antidepressants, antiparkinson drugs, antiepileptic drugs, and anxiolytics. Apart from antipsychotics and some antiparkinson drugs, many of these drugs do not mention NMS risks in their labels. Furthermore, when using favipiravir in adult patients, it is necessary to pay attention to NMS. In children, additional attention should be given to the adverse reactions of antiepileptic drugs and benzodiazepines.

NMS is a rare but potentially fatal adverse drug reaction. Previously published studies related to NMS are very limited and most of the information comes from case reports. Due to its pathogenesis mainly involving excessive blockade of dopamine uptake, an increasing number of non-antipsychotic drugs are being reported in association with NMS. Therefore, real-world pharmacovigilance studies are of significant importance in improving drug safety information. Currently, there are only two NMS drug safety monitoring studies, targeting the Japanese population. Through single-drug signal analysis, Kyotani et al. found that drugs related to NMS are primarily antipsychotics, as well as other non-antipsychotic medications, including antidepressants and antiparkinson drugs. In addition, they suggested that various pathways related were mainly neuroactive ligand-receptor interactions, dopaminergic synapses, or serotonergic synapses (Kyotani et al., 2023). Hirofuji et al. conducted disproportionality analysis and hierarchical cluster analysis for antipsychotic drugs, revealing stronger safety signals for haloperidol, chlorpromazine, risperidone, and aripiprazole. They also concluded that typical and atypical antipsychotic drugs exhibit different clinical manifestations related to NMS (Hirofuji et al., 2023). The FAERS reports are sourced from global data, encompassing a more diverse population dataset. To our knowledge, this is the first drug adverse reaction analysis targeting NMS based on the FAERS database, aiming to assist healthcare professionals in understanding post-market safety information of medications and managing NMS from the perspective of drug selection.

Among the top 50 drugs associated with NMS, antipsychotic medications, including both typical antipsychotics and atypical antipsychotics, dominate in terms of report count and safety signal strength rankings, which is consistent with previous studies (Hirofuji et al., 2023; Singhai et al., 2019). Although the pathophysiology of NMS is incompletely understood, the most widely held hypothesis is that NMS symptoms seem related to a rapid decrease in central dopaminergic activity because of the blockade of D2 receptors or the abrupt cessation of D2 receptor stimulation. The clinical manifestations can be explained as follows: The reduction in central dopaminergic neurotransmission in the striatum and hypothalamus leads to impaired thermoregulation. Blockade of striatal dopamine receptors contributes to muscle rigidity and tremor. Hypothalamic and spinal dopamine receptor antagonism results in altered mental status (Berloffa et al., 2021; Ware et al., 2018). This hypothesis can also explain the close association between metoclopramide, which acts as a dopamine D2 receptor antagonist, and NMS (Kocyigit et al., 2017; Wittmann et al., 2016). Due to the reduced dopaminergic blockade and the antagonistic effects on 5-HT receptors of non-typical antipsychotic medications, NMS induced by atypical antipsychotics is characterized by lower incidence, lower clinical severity, and less mortality (Belvederi Murri et al., 2015; Stevens, 2008). On the other hand, abrupt discontinuation or rapid switching of dopaminergic drugs that act as D2 receptor stimulation for Parkinson’s disease may precipitate NMS (Waqas et al., 2023; Wei and Chen, 2014). In line with case reports, this study categorizes carbidopa, bromocriptine, entacapone, amantadine, pramipexole, and others as suspected drugs for NMS,which respective labels also warn of the risk of NMS with dosage reduction and discontinuation, leading us to speculate that NMS occurrences in these reports occurred following dosage adjustment of these drugs.

Interestingly, an unexpected significant signal was identified with favipiravir. In this pharmacovigilance study, all 4 reported indications were COVID-19 infections. There is little knowledge about NMS related to COVID-19 infection. Reviewing the literature, only 6 case reports of NMS in COVID-19 patients have been published globally, with only 2 case reports involving patients using favipiravir (Borah et al., 2021; Durbach et al., 2022; Espiridion et al., 2021; Gökçen and Akkuş, 2024; Kajani et al., 2020; Soh et al., 2020). According to Soh et al., two COVID-19 patients diagnosed with NMS experienced a rapid reduction in elevated CK levels, a gradual resolution of fever, and stabilization of breathing following the discontinuation of favipiravir (Soh et al., 2020). Considering that these patients were also concurrently taking antipsychotic medications, we speculate that favipiravir may have influenced the metabolism of these medications to some extent. Its inhibitory effect on cytochrome P450 could disrupt the dopamine system, leading to neurotransmitter imbalance and potentially promoting the onset of NMS. However, there is currently insufficient evidence to fully explain the mechanism linking favipiravir to NMS. Based on the research findings, a causal relationship between favipiravir and NMS cannot be established. Firstly, coronaviruses are known for their neurotropic properties, which can lead to neurological and psychiatric symptoms ranging from peripheral to central nervous system involvement. In reported cases, some COVID-19 patients did not receive favipiravir, suggesting that the impact of COVID-19 on the central nervous system could increase susceptibility to the development of NMS. Furthermore, in the aforementioned cases, patients were also taking medications such as risperidone alongside favipiravir, and these medications were discontinued immediately following the onset of NMS. Therefore, the potential influence of antipsychotic drugs cannot be ruled out. In a word, when antipsychotic and anti-viral treatment is needed during any infection, especially in COVID-19, the risk of NMS should be taken into consideration.

Currently, there are some case reports about antidepressants triggering NMS (Garcia et al., 2001; Janati et al., 2012). In this study, antidepressants demonstrated a significant safety signal. The antidepressants included in the analysis comprise selective serotonin reuptake inhibitors (SSRIs), serotonin-norepinephrine reuptake inhibitors (SNRIs), noradrenergic and specific serotonergic antidepressants (NaSSAs), as well as tricyclic antidepressants (TCAs). NMS may be associated with a dysregulation of the dopamine and serotonin systems. Antidepressants may potentially inhibit the release of dopamine by increasing serotonin levels, thereby affecting the development of NMS. Spivak et al. measured eight NMS patients and found that dopamine concentrations were significantly lower during acute NMS states, while serotonin concentrations and the serotonin/dopamine ratio tended to be higher (Spivak et al., 2000). If antidepressants were used in combination with antipsychotics, it may exacerbate the antipsychotic-induced dopamine depletion, further increasing the risk of NMS. Additionally, considerations need to be given to the pharmacokinetic factors in the occurrence of NMS. For example, paroxetine may increase the blood concentration of antipsychotic drugs by inhibiting the metabolism of drugs such as risperidone through CYP2D6 inhibition (Stevens, 2008).

This study also evaluated potential differences in NMS reporting based on sex and age. As described in the baseline profile, males comprised the majority of reported NMS submitted to the FDA, which aligns with prior literature indicating a higher incidence of NMS among males (Gurrera, 2017). One possible reason may be the difference in the incidence of mental and neurological disorders between genders. Diseases like schizophrenia and Parkinson’s disease are more common in males, leading to males being more likely to be prescribed antipsychotics and antiparkinson drugs, thus increasing the risk of NMS (Bergen et al., 2014; Lubomski et al., 2014). In gender subgroup analysis, this study observed similar drug classes for signal strength in both males and females, which included antipsychotics, antiparkinson drugs, and antidepressants. However, in age subgroup analysis, the study found additional safety signals for antiepileptic drugs and benzodiazepines in the pediatric group compared to the adult group. Similarly, due to the different disease spectra of mental and neurological disorders, the types of medications used cannot be entirely consistent between pediatric and adult patients. In the indications recorded in this study, the proportion of pediatric epilepsy is higher than that in adults. Benzodiazepines are recommended for treating NMS, used for sedation and reducing peripheral muscle tone. There are literature reports of several cases developing NMS-like symptoms during withdrawal from benzodiazepines, hence considering a potential association between benzodiazepine withdrawal and NMS (Bobolakis, 2000; Kishimoto et al., 2013). The exact mechanism for how NMS is associated with antiepileptic is not yet completely understood. However, literature suggest that the co-administration of carbamazepine with tricyclic antidepressants and lamotrigine with antipsychotics may contribute to the occurrence of NMS, possibly due to the impact of antiepileptic drugs on the release of γ-aminobutyric acid (GABA) (Janati et al., 2012; Szota et al., 2020).

The current study has some strengths. First, FAERS is one of the largest public pharmacovigilance databases, with a sample size large enough to detect rare adverse events which would be difficult to detect in traditional epidemiological studies. Second, this study conducted subgroup analysis on different gender and age groups, providing essential insights for personalized medication management for different subgroup populations. Meanwhile, this study also has certain limitations. First, statistically detected signals cannot identify the causality between drugs and NMS. Second, due to limitations in the proactive, accurate, and timely reporting of adverse events by physicians and other healthcare providers, there may be possibilities of under-reporting and misreporting. Third, this study focuses on the safety signal analysis of a single drug. Due to the limitations of the database, further analysis of treatment regimens and drug dosage adjustments could not be conducted. However, in clinical practice, adjustments to medications and combination therapies may impact the occurrence of adverse events. Future research needs to further refine the analysis of these risk factors.

## 5 Conclusion

In conclusion, the present study comprehensively assessed NMS reports and associated drugs using the FAERS database. In addition to the known antipsychotic drugs, we detected significant safety signals related to NMS with non-antipsychotic medications such as antidepressants, antiparkinson drugs, and antiepileptic drugs. Also, future prospective clinical trials and epidemiologic investigations are needed to investigate the causal relationship between these drugs and NMS.

## Data Availability

The datasets presented in this study can be found in online repository: https://fis.fda.gov/extensions/FPD-QDE-FAERS/FPD-QDE-FAERS.html.
